# Evaluation of functionality for serine and threonine phosphorylation with different evolutionary ages in human and mouse

**DOI:** 10.1186/s12864-018-4661-6

**Published:** 2018-06-04

**Authors:** Benpeng Miao, Qingyu Xiao, Weiran Chen, Yixue Li, Zhen Wang

**Affiliations:** 1 Key Lab of Computational Biology, CAS-MPG Partner Institute for Computational Biology, Shanghai Institutes for Biological Sciences, Chinese Academy of Sciences, Shanghai, People’s Republic of China; 2 University of Chinese Academy of Sciences, Beijing, People’s Republic of China; 3 School of Life Science and Technology, Tongji University, Shanghai, People’s Republic of China; 4 Shanghai Center for Bioinformation Technology, Shanghai Industrial Technology Institute, Shanghai, People’s Republic of China; 5 Collaborative Innovation Center for Genetics and Development, Fudan University, Shanghai, People’s Republic of China

**Keywords:** Phosphorylation, Evolution, Function

## Abstract

**Background:**

Rapid evolution of phosphorylation sites could provide raw materials of natural selection to fit the environment by rewiring the regulation of signal pathways. However, a large part of phosphorylation sites was suggested to be non-functional. Although the new-arising phosphorylation sites with little functional implications prevailed in fungi, the evolutionary performance of vertebrate phosphorylation sites remained elusive.

**Results:**

In this study, we evaluated the functionality of human and mouse phosphorylation sites by dividing them into old, median and young age groups based on the phylogeny of vertebrates. We found the sites in the old group were more likely to be functional and involved in signaling pathways than those in the young group. A smaller proportion of sites in the young group originated from aspartate/glutamate, which could restore the ancestral functions. In addition, both the phosphorylation level and breadth was increased with the evolutionary age. Similar to cases in fungi, these results implied that the newly emerged phosphorylation sites in vertebrates were also more likely to be non-functional, especially for serine and threonine phosphorylation in disordered regions.

**Conclusions:**

This study provided not only insights into the dynamics of phosphorylation evolution in vertebrates, but also new clues to identify the functional phosphorylation sites from massive noisy data.

**Electronic supplementary material:**

The online version of this article (10.1186/s12864-018-4661-6) contains supplementary material, which is available to authorized users.

## Background

Genetic variations are the primary sources that contribute to the evolution of new phenotypes [1–3]. With the great advances in the high-throughput genome sequencing, substantial genomic variations across different species were characterized [4–7]. However, how the variations positively influence the phonotypic outcomes remains largely unexplored. Changes of protein phosphorylation, which is the most ubiquitous post-translational modification conducting cellular signals, have been drawing many attentions [8–10]. Although phosphorylation sites (phosphosites) are on average more conserved than non-phosphorylated counterparts in evolution, they exhibit rapid divergence among species due to their structural preference in disordered regions [11–15]. The high diversity of phosphosites would possibly rewire the cellular signaling transduction and response to environment, providing potential materials that natural selection could act upon. Based on the observation that genetic interactions between kinases and substrates were altered at a higher rate than average genes among three species of yeast, it was postulated that the evolution of phosphorylation regulation made crucial contribution to phenotypic fitness just as transcriptional regulation [2].

Nonetheless, another pervasive consequence of the rapid turnover of phosphosites would be that a large percentage of phosphosites are non-functional [16, 17]. Though there is little empirical evidence, it is possible in principle because of the limited specificity of kinases. This hypothesis was primarily supported by the fact that a large proportion (about 65%) of phosphosites with no characterized function evolved at a similar rate compared with non-phosphosites in disordered regions [16, 17]. Moreover, the evolutionary conservation of phosphosites was positively associated with phosphorylation stoichiometry but negatively associated with protein abundance in yeast, which could be explained by more accidental phosphorylation in abundant proteins [18]. A recent study presented a more comprehensive landscape of phosphorylation across 18 fungal species [19]. Through tracking the phylogeny of the phosphosites, the study revealed that while only about 2% of the phosphosites could be preserved longer than 700 million years, 69% were gained younger than 18 million years. Especially, relatively to the recently acquired phosphosites, the ancient ones are more likely to be functionally important, which implies the enrichment of noisy phosphorylation in the former.

The prevalence of young phosphosites with silent functions was observed in fungi, but the evolutionary pattern in more complex vertebrate species was elusive. After all, the sophisticated development program and communications among numerous cells in vertebrate species strongly depend on signaling pathways mediated by phosphorylation [10, 20–24] . In fact, it was shown that phosphosites involved in vertebrate-specific functional modules (VFMs) were even more conserved than those in basic functional modules (BFMs) [25]. In this study, with the large-scale data of human and mouse phosphosites, we aimed to evaluate the functionality of phosphosites with different evolutionary ages across vertebrates.

## Results

### Three evolutionary age groups of phosphosites

**Fig. 1:**
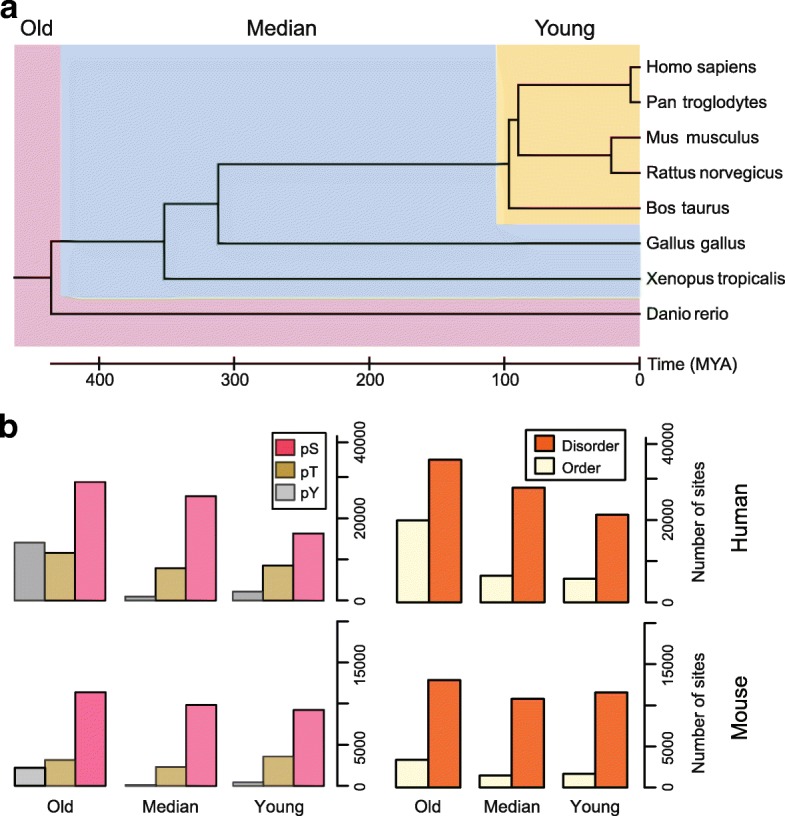
Three evolutionary age groups of phosphosites**.** (**a**) The age groups of phosphoites were divided in to the old, median and young based on the phylogeny of 8 vertebrate species. (**b**) The distributions of three kinds of phosphosites and local structures in the age groups. pS: phosphor-serine; pT: phosphor-threonine; pY: phosphor-tyrosine

Most of phosphorylation data on vertebrates were generated from model organisms, such as human and mouse [15, 26]. Due to the unbalance of phosphorylation studies, it was infeasible to directly compare the phosphorylated status across different species [19]. However, as is known that the appearance of phosphor-acceptor amino acids (serine, threonine or tyrosine) was essential for phosphorylation [8, 27], it was reasonably assumed that the later appearance time of those amino acids in evolution would represent younger phosphorylated status. In this study, we separately collected 196,093 human and 62,274 mouse phosphosites from the PhosphoSitePlus database (version: 2015.12) [26], and 5747 phosphosites were shared by the two species (Additional file 1: Figure S1). We reconstructed the ancestral sequences of the phosphorylation proteins across 8 vertebrates (see Methods). Based on the first appearance time of the phosphor-acceptor amino acids in the vertebrate species tree, we divided the phosphosites into three different age groups: old, median and young (Fig. 1a). In the old group, the phosphosites kept conserved across all the vertebrates and emerged earlier than 435 million years ago (MYA), whereas the phosphosites in the young group were generated during the divergence of the mammal species (about 96 MYA) (Fig. 1a). The phosphosites with evolutionary age between the old and young group were defined to the median group.

The basic characters of phosphosites in the three age groups were analyzed. For the three phosphor-acceptor amino acids, the serine and threonine showed relatively balanced distribution among the age groups, while the tyrosine was much more enriched in the old group (Fig. 1b). As the difference in bio-chemical properties of the serine/threonine and tyrosine and that they were phosphorylated by different types kinases, our results indicated their different evolutionary dynamics. In each age group, there were more phosphosites located in disordered regions than in the ordered regions, consistent with the knowledge that they were more likely to be recognized by kinases on the surface of protein [14, 15, 28, 29] (Fig. 1b). In the following analyses, we mainly focused on the phosphosites of serine/threonine in the disordered regions, from which most of the evolutionary diversity resulted across the species .

### Functional annotations of phosphosites in different evolutionary age groups

**Fig. 2:**
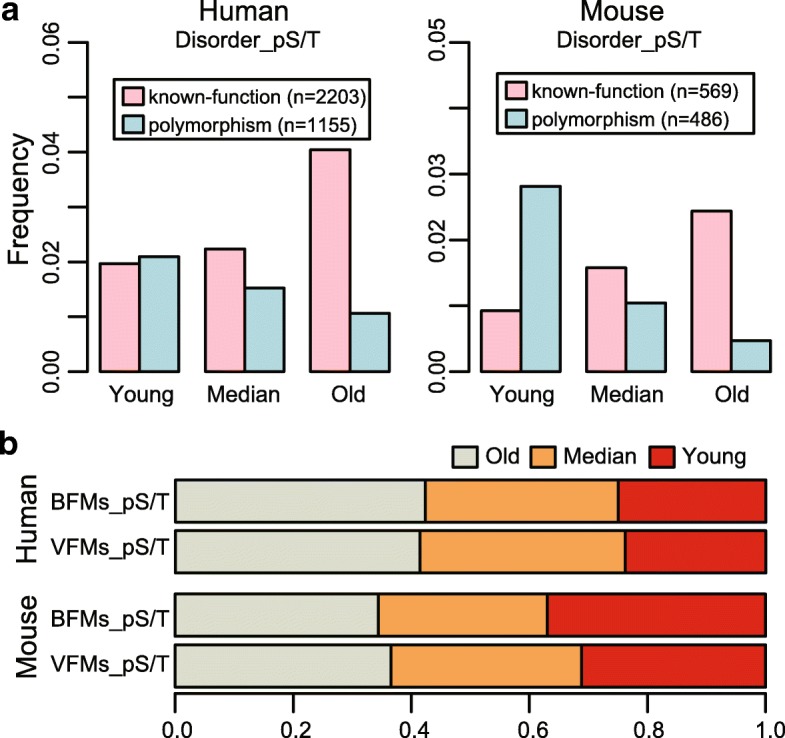
Functional annotations of phosphoites across age groups. (**a**) The known-functional and polymorphic sites in disordered regions were differently distributed among the three age groups for human and mouse. The functional phosphosites were more likely to be older and polymorphic sites were more likely to be younger (*p*-value< 0.01, Chi-squared test). (**b**) The proportion of age groups in disordered regions varied between BFMs and VFMs. In both human and mouse, young phosphosites occupied larger proportion in the BFMs than the VFMs (p-value = 0.035 and p-value< 0.01, separately, Chi-squared test). BFMs: basic functional modules; VFMs: vertebrate-specific functional modules

To evaluate the functional potential of phosphosites in different age groups, we took several types of annotations into consideration. Firstly, we separately gathered human and mouse known-functional phosphosites from the PhosphoSitePlus database and polymorphic phosphosites in populations from dbSNP [26, 30] (See Methods). We compared the proportion of the known-functional and polymorphic phosphosites among the three age groups, respectively. For both human and mouse, the proportion of known-functional phosphosites was significantly raised with increase of the evolutionary age (human: *p*-value < 0.01, mouse: *p*-value < 0.01, Chi-squared test), while the proportion of the polymorphic phosphosites was significantly decreased with the increase of the evolutionary age (human: *p*-value < 0.01, mouse: *p*-value < 0.01, Chi-squared test, Fig. 2a). The trends were also significantly observed for both human tyrosine phosphosites and serine/threonine phosphosites in ordered regions (Additional file 1: Figure S2). These results were well in agreement with the cases in yeasts, which suggested that functional phosphosites in vertebrates tended to be older in evolution. Meanwhile, as polymorphic sites were more likely to be evolutionarily neutral, our results implicated that a higher proportion of young phosphosites might be non-functional.

Next, due to the limited functional annotation of phosphosites, we took the proteins where the phosphosites resided into consideration. Wang et al. divided phosphorylated proteins into two broad modules: basic functional modules (BFMs) that shared by both vertebrates and non-vertebrates, and vertebrate-specific functional modules (VFMs) [25]. In this study, it was shown that the phosphosites in VFMs showed higher evolutionary conversation than those in BFMs, which was validated in both the human and mouse (*p*-value = 1.50e-09 and *p*-value < 2.2e-016, separately, Wilcox rank sum test, Additional file 1: Figure S3A). In particular, there was a larger percentage of young phosphosites distributed in the BFMs compared with the VFMs (human: *p*-value = 0.035, mouse: *p*-value = 9.17e-10, Chi-squared test, Fig. 2b). In addition, a smaller proportion of young phosphosites in the BFMs were known-functional compared with those in the VFMs (human: *p*-value = 5.63e-07, mouse: *p*-value = 1.87e-07, Fisher exact test, Additional file 1: Figure S3B). As the VFMs contained many signaling pathways related proteins in which functional phosphosites were more likely to be involved, the results also implied that phosphosites in the young group were less functionally important than those in the old group.

### Enrichment analysis of ancestral state for the phosphosites

**Fig. 3:**
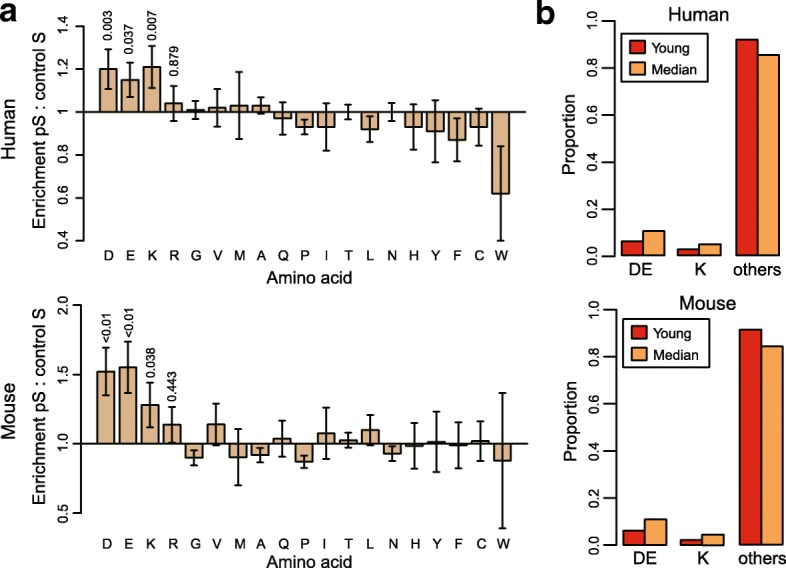
Analysis of ancestral state of phosphosites. **a** Enrichment analysis of amino acids in the transition to phosphosites in disordered regions for human and mouse. The labs of x-axis were the abbreviation of amino acids. Comparing with control data, three kinds of amino acids (D, E and K) were enriched in the transition to phosphosites in both human and mouse. **b** The distribution of phosphosites evolved from different amino acids in the median and young groups. In both human and mouse, there were more phosphosites evolving from D/E in the median group than the young group (p-value< 0.01, Chi-squared test). D: Aspartate; E: Glutamate; K: Lysine

It has been reported that more phosphosites could evolve from aspartate/glutamate (Asp/Glu) residues than corresponding non-phosphosites significantly [31, 32]. As the unpredictability of ancestral state for the old group conserved across all the vertebrates, we only considered phosphosites in the median and young groups for the analysis. Following Pearlman et al. [31], we did the enrichment analysis of the ancestral state for the phosphosites, taking the corresponding non-phosphorylated residues in the same age groups as the control (see Methods). In both human and mouse datasets, we observed significantly more phosphor-serine with evolutionary transition from ancestral Asp/Glu residues in the disordered regions (Fig. 3a). However, the enrichment was not significant for phosphor-threonine, phosphor-tyrosine and those in ordered regions, which probably due to the small sample size (Additional file 1: Figures S4 and S5). In addition to those results, we also discovered that lysine (Lys), a positively charged amino acid, was enriched in the transition to phosphor-serine, probably due to the fact that phosphorylation could also act upon Lys [33–35].

Asp/Glu, the negatively charged amino acids, can sometimes mimic the function of the phosphorylated residues with negative charge [31, 36, 37]. Thus, it was assumed that the phosphorylation of the residues evolved from Asp/Glu could activate the ancestral function of proteins. Consistent with the assumption, we found that there was a significant higher proportion of phosphosites transited from Asp/Glu distributed in the median group than in the young group (human: *p*-value < 2.2e-16, mouse: *p*-value < 2.2e-16, Chi-squared test, Fig. 3b). We also observed the similar results for the phosphosites transited from Lys (human: *p*-value < 2.2e-16, mouse: *p*-value < 2.2e-16, Chi-squared test). These provided another evidence that younger phosphosites of serine in disordered regions were more likely to be non-functional.

### Phosphorylation level and breath among tissues

As reported that in yeasts, phosphosites of higher stoichiometry were more conserved and functional [18, 38]. However, the relationship among quantitative features and evolutionary conservation was not systematically studied in the different tissues of vertebrates. In this study, we collected 14,954 mouse phosphosites with phosphorylation levels across nine different tissues [39]. Then, we compared the maximum phosphorylation level and phosphorylation breadth (i.e., number of tissues where the phosphorylation was expressed) among the three age groups (see Methods). Meanwhile, as the quantitative features of phosphosites might be confounded by those of the whole protein, we excluded the bias resulted from protein abundance and breadth.

**Fig. 4:**
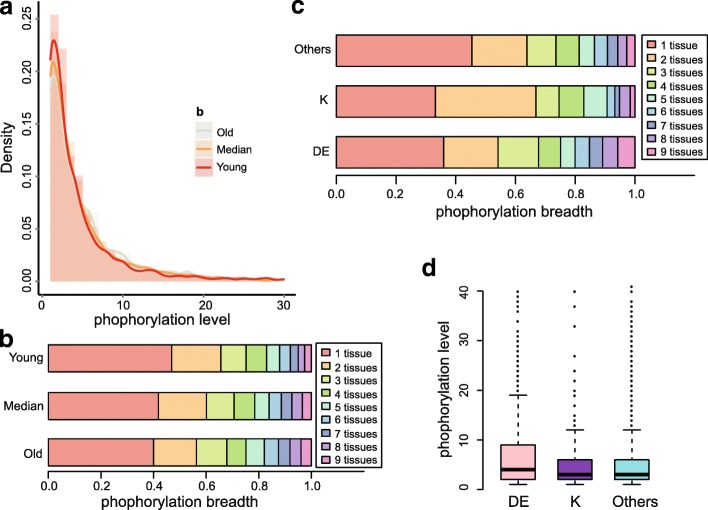
Phosphorylation level and breadth in mouse. (**a**) The distribution of maximum level for phosphosites in the three age groups. The phosphorylation level was lower in the young group than the old group (p-value = 1.87e-10, Wilcox rank sum test). (**b**) The phosphorylation breadth in the three age groups. Higher proportion of phosposites in the young group expressed in fewer tissues compared with the old group (p-value = 7.52e-12, Wilcox rank sum test). (**c**) The phosphorylation breadth of phosphopsites evolving from different amino acids. Larger part of phosphosites evolving from DE expressed in more tissues than other kind of phosphosites. (**d**) The maximum phosphorylation level for phosphosites originating from different amino acids. There were more phopshosites originating from DE with high phosphrylation level than other kinds of phosphosites

We identified that the maximum phosphorylation level in the young group was lower compared with the old group (*p*-value = 1.87e-10, Wilcox rank sum test, Fig. 4a), but the protein abundance between the two groups showed no difference (*p*-value = 0.41, Wilcox rank sum test, Additional file 1: Figure S6A). We also found that the phosphorylation breadth was lower in the young group than the old group (*p*-value = 7.52e-12, Wilcox rank sum test, Fig. 4b). Although the protein expression breath showed difference (*p*-value < 2.2e-16, Wilcox rank sum test, Additional file 1: Figure S6B), the ANONA analysis suggested that the contribution of the phosphorylation breadth could not be compensated (*p*-value < 0.01) taking into account the two factors simultaneously. Furthermore, there was a larger proportion of phosphosites with both low phosphorylation level and breadth in the young group compared with the old group (*p*-value = 5.1e-04, Chi-squared test, Additional file 1: Figure S7). And with the stepwise regression, the contribution of phosphorylation level could be compensated by the breadth, indicating the high correlation between phosphorylation level and breadth (*r* = 0.46, Pearson’s correlation). These results indicated that with the increase of evolutionary age, both the phosphorylation level and breadth tended to be increased, which could be more likely to keep essential functions.

It was interesting that phosphosites evolved from Asp/Glu displayed higher phosphorylation level and breadth than those originated from other amino acids (*p*-value = 1.89e-08 and *p*-value = 1.12e-05, separately, Wilcox rank sum test, Fig. 4C and D). This supported our argument that this type of phosphosites was more intended to be functional. However, this trend was not observed for the phosphosites originated from Lys (*p*-value = 0.31 and *p*-value = 0.24, separately, Wilcox rank sum test). Thus, quantitative features would be also helpful to prioritize functional phosphosites.

## Discussion

As with genetic variants, rapid changes of protein phosphorylation would play important roles in the evolution of new phenotypes. However, a large part of phosphosites was speculated to be non-functional due to the non-specific recognition of kinases [16, 17]. Computational methods integrating evolutionary conservation, structural preference and kinase recognition were proposed to explore functional phosphosites from large-scale data [40–42]. And a recent fungi study showed that young phosphosites prevailed and contributed to the major noisy of phosphorylation [19]. In this study, we investigated whether the evolutionary age of phosphosites was also associated with their functionality in vertebrates by dividing the phosphosites into old, median and young groups based on their emergence time in the species tree.

We found several evidences that the functional potential of phosphosites was increased with their evolutionary age especially for serine and threonine in the disordered regions, which was separately proved in the human and mouse data. Firstly, the old group harbored a higher proportion of known-functional phosphosites and a lower proportion of polymorphic phosphosites than the young group. Secondly, in VFMs where phosphorylation was more essential, there was a lower fraction of young phosphosites than in BFMs. Thirdly, fewer phosphosites originating from Asp/Glu which could mimic their ancestral functions, were evolutionarily young. Finally, the phosphorylation level and breadth among different tissues were increased with evolutionary age. And the phosphorylation level is highly correlated with the breadth.

Consistent with the study in fungi, our results provided a comprehensive scenario for the evolution of phosphorylation. The high turnover rate in disordered regions facilitated the birth of new phosphosites. However, most of them would be non-functional and eliminated due to the lack of functional constrains. The rapid ‘try-and-error’ process provided potential materials for the innovation of phenotypic fitness, and only those phosphosites acquiring indispensable functions could be retained during the long evolutionary time. The scenario provided a new approach to screen out the functional ones from massive phosphosite data by a combination of several factors, including evolutionary age, ancestral state, phosphorylation level and breadth.

It is important to note that the insights of tyrosine phosphorylation and the phosphorylation in ordered regions were limited in our study. This was because that the evolutionary rates of the two types were much lower than the serine and threonine phosphorylation in disordered regions, leading that only a small amount of data was useful. However, it was also observed that for both tyrosine phosphosites and serine/threonine phosphosites in ordered regions, fewer known-functional and more polymorphic phosphosites were distributed in the young group, indicating that they were subjected to the similar pattern. Another possible concern was that we identified the ages based on the phylogeny of phospho-acceptor, which was an upper bound for the emergence of phosphorylation status. For example, it was possible that some phosphosites in the old group might be phosphorylated in recent time. Therefore more comprehensive phosphorylation datasets covering different species were necessary to infer the phosphorylation age more precisely.

## Conclusions

In summary, the current study evaluated the functionality of human and mouse phosphosites, indicating that newly emerged vertebrate phosphosites were more likely to be non-functional, especially for serine and threonine phosphorylation in disordered regions. Meanwhile, our study provided a new evolutionary scenario of phosphorylation. New phosphosites were caused by the high turnover rate in disordered regions, and only those with indispensable functions could result in the innovation of phenotypic fitness during the long evolutionary time. We also provided useful insights to screen out the functional ones from massive phosphosite data by a combination of the evolutionary age, ancestral state, phosphorylation level and breadth.

## Methods

### Classification of Phosphosites

One hundred ninety-six thousand ninety-three human phosphosites of 16,031 proteins and 62,274 mouse phosphosites of 9600 proteins were gathered from the PhosphoSitePlus database (version: v2015.12) separately [26]. To classify the phosphosites by the evolutionary ages, 8 vertebrate species were selected, including *H. sapiens, P. troglodytes, M. musculus, R. norvergicus, B. taurus, G. gallus, X. tropicalis* and *D. rerio.* We built the species tree and got the divergence time from TimeTree.org [43]. The orthologous proteins of these species were found from InParanoid8, and multiple sequence alignments were performed with Clustal-omega [44, 45]. Then, the FastML.v3.1 software was used to reconstruct the ancestral sequences of phosphorylation proteins [46, 47]. The phophosites were filtered by the following rules: the number of orthologous sequences should be more than 4 to infer the ancestral state. Finally, we got 115,780 human phosphosites and 42,244 mouse phosphosites respectively (Additional file 1: Table S1).

### Local structure

The VSL2 software was used to identify the phosphosites in ordered or disordered regions [48]. It calculated the disorder score (between 0 and 1) for each amino acid based on 26 amino acids-based features for a given protein sequence. The score of phosphosites in disordered regions should be greater than 5. Otherwise, the phosphosites were in ordered regions.

### Functional annotation

The human and mouse functional phosphosites were collected from a file named ‘Regulatory_sites.gz’ in the PhosphoSitePlus database (file name: Regulatory_sites, version: v2015.12) [26]. We respectively got human and mouse polymorphic phosphosites by overlapping with human and mouse SNPs annotated from dbSNP138 using ANNOVAR (version: 20150619, GRCH38 and GRCM38) [49]. The definition of BFMs and VFMs for phosphorylation proteins refered to Wang et al. [25]. And Rate4sites [50] was used to calculate the evolutionary rate of phosphosites and compared the rates between BFMs and VFMs.

### Enrichment of ancestral state

We explored whether phosphosites in the median and young group could significantly originate from some amino acid residues compared with corresponding non-phosphosites. The ancestral state of those sites was retrieved from the FastML.v3.1 results. For the control dataset, we collected serine, threonine and tyrosine sites which were not known to be phosphorylated from the same proteins within the age groups. We filtered the control dataset was filtered by the following criterion: 1) the sites overlapped with the verified and predicted phosphosites from dbPTM were excluded; 2) we eliminated the potential phosphosites predicted by Networkin with the score greater than 2 were eliminated. According to the method of Pearlman et al., we calculated the ratio and *p*-value of enrichment for each ancestral amino acid by bootstrap sampling with 1000 times. For every sampling, the distribution of phosphosites in disordered regions was balanced between phosphosites and control datasets.

### Phosphorylation quantification

Fourteen thousand nine hundred fifty-four mouse phosphosites with quantification data were gathered in nine tissues [39]. Overlapping with the age groups in disordered regions, we respectively got 2947, 2359 and 2310 sites in the old, median and young groups. Among the age groups, we respectively compared the maximum phosphorylation level across the tissue and the number of tissues where the phosphosites were expressed. In order to exclude the influence of protein abundance and breadth, the ANOVA analysis was performed ting both the phosphorylation and protein quantification into account.

## Additional files

Additional file 1: **Figure S1.** The shared phosphosites between human and mouse. **Figure S2.** The functional annotations for human tyrosine phosphosites and serine/threonine phosphosites in ordered regions. **Figure S3.** The difference of conservation and functional annotations between BFMs and VFMs. **Figure S4.** Enrichment analysis of amino acids in the transition to phosphosites for human. **Figure S5.** Enrichment analysis of amino acids in the transition to phosphosites for mouse. **Figure S6.** The distribution of protein abundance and breath in mouse. **Figure S7.** The phosphosites with both low phosphorylation level and breadth significantly enriched in young group compared with old group. **Table S1.** The number distribution of human and mouse phosphosites. (DOCX 226 kb)

## Abbreviations

phosphosites: phosphorylation sites
VFMs: vertebrate-specific functional modules
BFMs: basic functional modules
MYA: million years ago
Asp: aspartate
Glu: glutamate; Lys: lysine.
