# Supplementary material for: Evaluation of functionality for serine and threonine phosphorylation with different evolutionary ages in human and mouse
Source: BMC Genomics. 2018 Jun 4;19:431. doi: 10.1186/s12864-018-4661-6 (PMC5987384; doi:10.1186/s12864-018-4661-6)
Supplement: Supplementary file 1 — Figure S1. The shared phosphosites between human and mouse. Figure S2. The functional annotations for human tyrosine phosphosites and serine/threonine phosphosites in ordered regions. Figure S3. The difference of conservation and functional annotations between BFMs and VFMs. Figure S4. Enrichment analysis of amino acids in the transition to phosphosites for human. Figure S5. Enrichment analysis of amino acids in the transition to phosphosites for mouse. Figure S6. The distribution of protein abundance and breath in mouse. Figure S7. The phosphosites with both low phosphorylation level and breadth significantly enriched in young group compared with old group. Table S1. The number distribution of human and mouse phosphosites. (DOCX 226 kb) [file 12864_2018_4661_MOESM1_ESM.docx]

SUPPLEMENTARY FIGURES


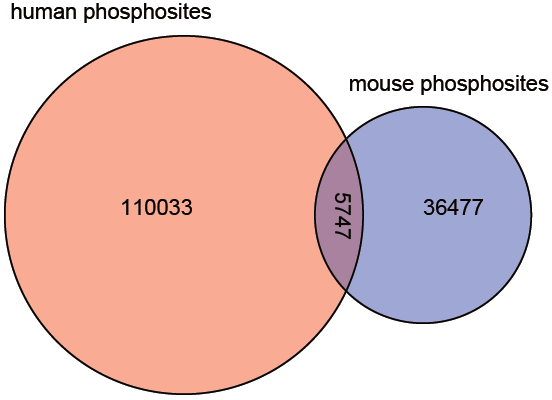


**Figure S1. The shared phosphosites between human and mouse.** There were 5,747 shared phosphosites, occupying about 5.0% and 13.6% in human and mouse datasets respectively.


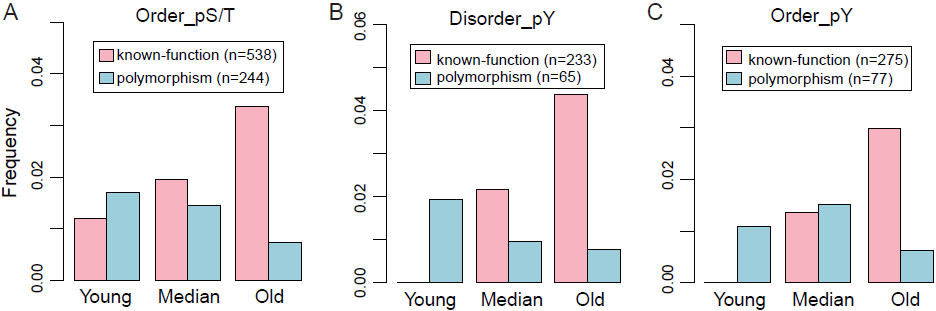


**Figure S2. The** **functional annotations for human tyrosine phosphosites and serine/threonine phosphosites in ordered regions.** (A) For serine/threonine phosphosites in ordered regions, the functional phosphosites were more likely to be older (p-value = 2.19e-13, Chi-squared test) and polymorphism sites significantly tended to be younger (p-value = 8.01e-08, Chi-squared test). (B-C) Compared with old group, the young group contained fewer known-function phosphotyrosine (disorder: p-value < 0.01, order: p-value < 0.01, Fisher's exact test) and more polymorphism phosphotyrosine (disorder: p-value = 0.0019, order: p-value = 0.082, Fisher's exact test). pS: phosphor-serine; pT: phosphor-threonine; pY: phosphor-tyrosine.


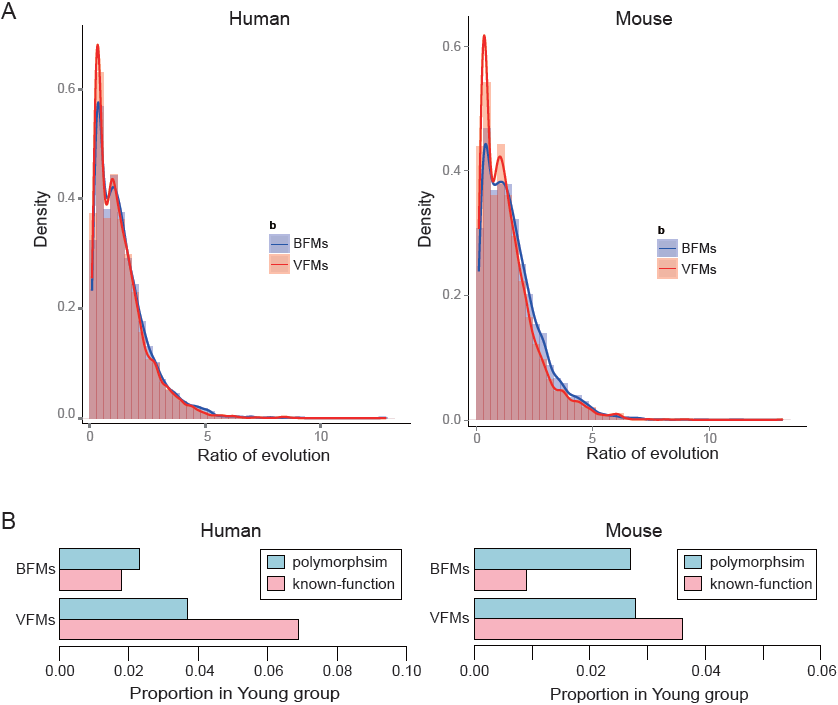


**Figure S3. The difference of conservation and functional annotations between BFMs and VFMs.** (A) The phoshosites in BFMs had higher evolutionary rate than the VFMs (human: p-value = 1.50e-09, mouse: p-value < 2.2e-016, Wilcox rank sum test). (B) The distributions of known-function and polymorphism phosphosites in Young group were different between BFMs and VFMs. The known-function phosphosites were more likely to enrich in the young phosphosites of VFMs compared with BFMs (human: p-value=5.63e-07, mouse: p-value=1.87e-07, Fisher exact test). But there was no difference of polymorphism between BFMs and VFMs (human: p-value= 0.18, mouse: p-value= 0.92, Fisher exact test). BFMs: Basic Functional Modules; VFMs: Vertebrate-specific Functional Modules.


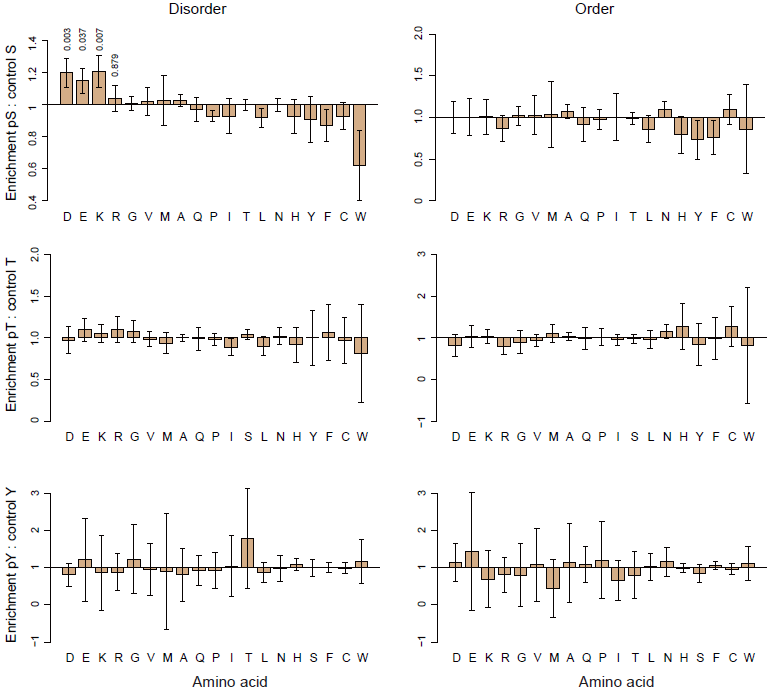


**Figure S4. Enrichment analysis of amino acids in the transition to phosphosites for human.** It was only observed that significantly more phosphor-serine in the disordered regions evolve from aspartate/glutamate residues than corresponding non-phosphosites.


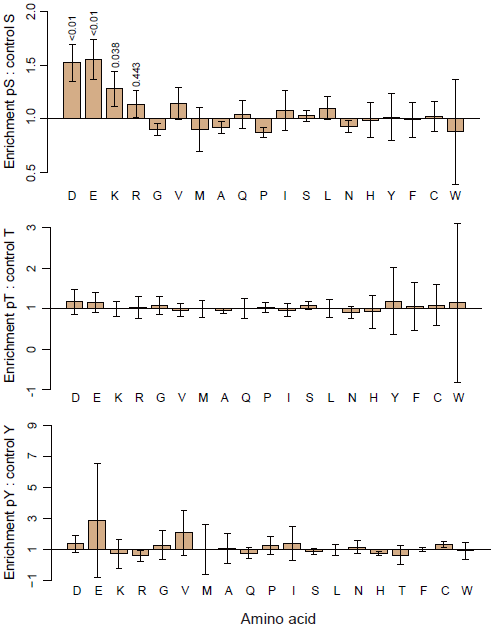


**Figure S5. Enrichment analysis of amino acids in the transition to phosphosites for mouse.** For phosphor-threonine and phosphor-tyrosine in disordered regions, there were not any amino acids enriched in the transition to phosphosites.


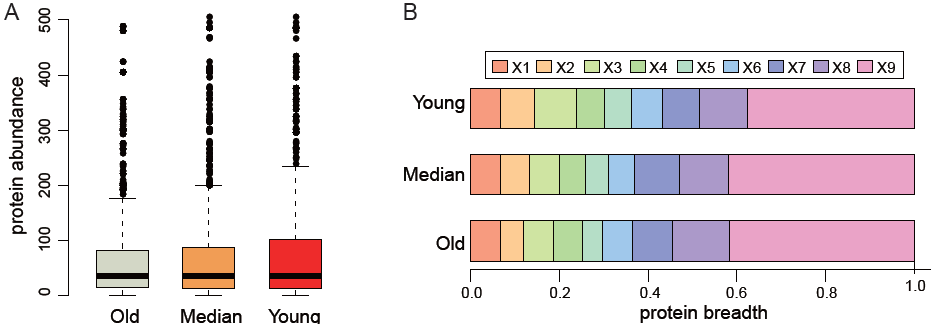


**Figure S6. The distribution of protein abundance and breath in mouse.** (A) There was no difference of protein abundance between old and young groups (p-value = 0.41, Wilcox rank sum test). (B) The distribution of protein breath between old and young groups was significantly different (p-value < 2.2e-16, Wilcox rank sum test).


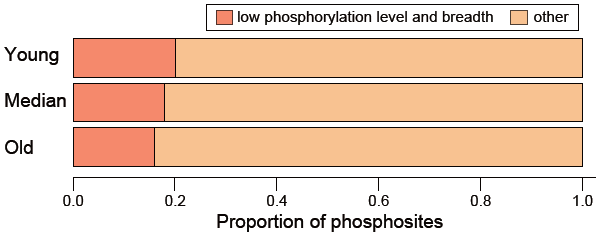


**Figure S7.** The phosphosites with both low phosphorylation level and breadth significantly enriched in young group compared with old group (p-value=5.1e-04, Chi-squared test). The low phosphorylation level and breadth was equal to 1 respectively.

SUPPLEMENTARY TABLES

**Table S1.** The number distribution of human and mouse phosphosites.

| Species | Total | Phosphorylation type | | Age group | |
| --- | --- | --- | --- | --- | --- |
| human | 115,780 | phosphor-serine | 70,478 | old | 54,501 |
|  |  | phosphor-threonine | 28,029 | median | 34,263 |
|  |  | phosphor-tyrosine | 17,273 | young | 27,016 |
| mouse | 42,224 | phosphor-serine | 30,448 | old | 16,744 |
|  |  | phosphor-threonine | 9,042 | median | 12,250 |
|  |  | phosphor-tyrosine | 2,754 | young | 13,230 |
